# Gas-phase endstation of electron, ion and coincidence spectroscopies for diluted samples at the FinEstBeAMS beamline of the MAX IV 1.5 GeV storage ring

**DOI:** 10.1107/S1600577520007146

**Published:** 2020-06-22

**Authors:** Kuno Kooser, Antti Kivimäki, Paavo Turunen, Rainer Pärna, Liis Reisberg, Marco Kirm, Mika Valden, Marko Huttula, Edwin Kukk

**Affiliations:** a Department of Physics and Astronomy, University of Turku, FIN-20014 Turku, Finland; b Institute of Physics, University of Tartu, W. Ostwaldi 1, EE-50411 Tartu, Estonia; c Nano and Molecular Systems Research Unit, University of Oulu, PO Box 3000, FIN-90014 Oulu, Finland; d MAX IV Laboratory, Lund University, PO Box 118, SE-22100 Lund, Sweden; e Surface Science Group, Laboratory of Photonics, Tampere University of Technology, FIN-33101, Tampere, Finland

**Keywords:** multi-particle coincidence spectroscopy, electron and nuclear dynamics, gas-phase endstation, VUV and soft X-ray spectroscopy, MAX IV

## Abstract

Since spring 2019 ▸ an experimental setup consisting of an electron spectrometer and an ion time-of-flight mass spectrometer for diluted samples has been available for users at the FinEstBeAMS beamline of the MAX IV Laboratory in Lund, Sweden. The setup enables users to study the interaction of atoms, molecules, (molecular) microclusters and nanoparticles with short-wavelength (vacuum ultraviolet and X-ray) synchrotron radiation and to follow the electron and nuclear dynamics induced by this interaction. Test measurements of N_2_ and thiophene (C_4_H_4_S) molecules have demonstrated that the setup can be used for many-particle coincidence spectroscopy. The measurements of the Ar 3*p* photoelectron spectra by linear horizontal and vertical polarization show that angle-resolved experiments can also be performed. The possibility to compare the electron spectroscopic results of diluted samples with solid targets in the case of Co_2_O_3_ and Fe_2_O_3_ at the Co and Fe *L*_2,3_-absorption edges in the same experimental session is also demonstrated. Because the photon energy range of the FinEstBeAMS beamline extends from 4.4 eV up to 1000 eV, electron, ion and coincidence spectroscopy studies can be executed in a very broad photon energy range.

## Abstract 2

An overview of the gas-phase endstation with the multi-coincidence setup at the FinEstBeAMS beamline in the MAX IV 1.5 GeV storage ring is presented. A detailed description of the functional design of the endstation together with the data acquisition concept and first test measurements is given.

## 1. Introduction   

The recent emergence of new light sources such as low-emittance storage rings, soft and hard X-ray free-electron lasers has stimulated studies of the electronic structure and dynamics of atoms, molecules, clusters and liquids, by photoelectron spectroscopy, ion spectroscopy and X-ray spectroscopy techniques (Piancastelli *et al.*, 2010 ▸; Berrah *et al.*, 2010 ▸).

Molecular photofragmentation is one of the important branches of studies concerning molecular reaction dynamics. The simplest example of photodissociation processes is a two-body reaction of diatomic molecules, in which a parent ion breaks into just two pieces. In this case the kinematic and energetic information about one single fragment is sufficient to acquire almost complete knowledge of the dynamics of the whole photodissociation event. Photoionization of more complex systems may lead to the creation of more than two fragments. In general, if the number of products from a photoionization event is *n*, the detection of motion characteristics of *n* − 1 products is needed to fully specify the dissociation dynamics. The multibody fragmentation processes can be studied by the photoionization in the vacuum ultraviolet (VUV) and X-ray radiation domain. The photoabsorption of VUV photons up to 20–30 eV usually causes single ionization while at higher photon energies multiple ionization becomes possible, producing positive ions, neutral fragments and electrons (photoelectrons and Auger electrons).

The detection and correlation of photoions and photoelectrons (or Auger electrons) from the same photoionization process can provide a dynamically complete description of the photofragmentation reactions (Arion & Hergenhahn, 2015 ▸). The energy analysis of the ejected electrons enables the determination of the initial electronic state of the created photoions. A time-of-flight (TOF) ion spectrometer equipped with a position-sensitive detector based on delay-line technology can record the TOF and impact position of each fragment ion on the detector. The data from the TOF spectrometer allows us to retrieve the final state momentum of the ion in all three spatial dimensions. This coincidence spectroscopy technique was first mentioned in 1987 (Eland, 1987 ▸) and nowadays photoelectron–photoion–photoion coincidence (PEPIPICO) spectroscopy and Auger electron–photoion–photoion coincidence (AEPIPICO) are important spectroscopic methods, especially since the introduction of multi-hit position-sensitive delay-line detectors (Jagutzki *et al.*, 2002*a* ▸,*b* ▸). An overview of alternative or supplementary experimental approaches in the area of coincidence spectroscopy was recently given by Arion & Hergenhahn (2015 ▸). Many setups have been built with different geometries for the electron analyzer [electron TOF spectrometer (Garcia *et al.*, 2013 ▸), magnetic bottle (Rademann *et al.*, 1991 ▸; Penent *et al.*, 2005 ▸), hemispherical analyzer (Ulrich *et al.*, 2011 ▸; Kugeler *et al.*, 2003 ▸; Kukk *et al.*, 2007 ▸), toroidal analyzer (Céolin *et al.*, 2004 ▸), COLTRIMS]. The COLTRIMS setups are extremely competitive for photoelectron–ion–ion coincidences due to the 4π acceptance angle (Prümper *et al.*, 2005 ▸; Sann *et al.*, 2016 ▸). However, the advantage of using a hemispherical electron analyzer in a coincidence setup over many other alternatives such as electron TOF spectrometer or COLTRIMS lies in its high and easily adjustable electron energy resolution in a broad kinetic energy range.

Here we describe the gas-phase coincidence spectroscopy endstation (GPES) of the FinEstBeAMS beamline in the 1.5 GeV storage ring at the MAX IV Laboratory in Lund, Sweden. The tunable photon energy range of this beamline (4.4–1000 eV) enables electron, ion and coincidence spectroscopic studies of the valence and core levels of a variety of sample systems such as molecules, clusters, nanoparticles and liquids. An overview of the experimental configurations of the GPES and its opportunities is given. The selected examples of measurements are not given as novel scientific results, but to illustrate the capabilities of the present setup.

## 2. Gas-phase branch at the FinEstBeAMS beamline   

### 2.1. Main characteristics of the FinEstBeAMS beamline   

The design and optical concept of the FinEstBeAMS beamline was described in detail in an earlier paper (Pärna *et al.*, 2017 ▸). The photon energy range of the FinEstBeAMS beamline extends from the ultraviolet to soft X-ray range (4.4–1000 eV) and a variable polarization of synchrotron radiation is possible. Such a wide energy range enables exploration of both valence and core level interaction with electromagnetic radiation. The polarization of the beam can be adjusted by an elliptically polarizing undulator [APPLE II design (Sasaki *et al.*, 1993 ▸)], which enables one to vary the polarization plane of linearly polarized radiation or to use an elliptically (circularly) polarized beam for experiments. The photon energy can be selected by a plane-grating monochromator (SX700 type, manufactured by FMB Feinwerk-und Messtechnik GmbH, Berlin, Germany), which has two side-cooled plane gratings (600 lines mm^−1^ and 92 lines mm^−1^). High orders of diffracted light at low photon energies can be eliminated by the combination of gas and thin film filters (only thin-film filters are presently installed). The spot size at the focus of the endstation is 100 µm × 100 µm. The overall resolving power is in the range 5000–10000. The highest photon energy resolution is about 1.2 meV at 21.58 eV and 30–40 meV at 401 eV. The photon flux in the photon energy range 50–100 eV stays above 10^13^ photons s^−1^ using a resolving power of 5000. Photon flux at 1000 eV is about 4 × 10^9^ photons s^−1^.

### 2.2. Gas-phase endstation (GPES) – design of the experimental sections   

The endstation with two separate experimental sections is designed and built for spectroscopic studies of diluted gas phase samples like molecules, (micro)clusters, free-standing nanoparticles as well as liquids. The free-standing nanoparticles are injected into the experimental chamber from the colloidal solution by atomizer or nebulizer system or by liquid micro-jet setup. The freestanding nanoparticles are not desorbed to any surfaces, but can be injected into the X-ray beam by carrier gas (usually noble gas) or by solvent liquid. In order to maintain ultra-high vacuum (UHV) conditions at the FinEstBeAMS beamline, the GPES is connected to the beamline via a differential pumping section, consisting of three stages (a 300 l s^−1^ turbomolecular pump, a line-of-sight differential ion pump from XIA, and an 80 l s^−1^ ion pump). These windowless pumping stages permit pressure differences between the beamline and GPES of up to five orders of magnitude, avoiding contamination of the beamline optics with gas phase samples from the experimental sections. The endstation is designed and planned for experimental techniques like high-resolution electron spectroscopy, ion TOF mass spectroscopy, velocity mapping and multiparticle coincidence spectroscopy. Also time-resolved studies can be conducted at the GPES but generally they require both single-bunch operation of the storage ring and a beam chopper (Förster *et al.*, 2015 ▸; Ito *et al.*, 2009 ▸). The first two experiments with single-bunch operation have already been performed at the FinEstBeAMS using a photoluminescence endstation and a magnetic bottle installed at the GPES (see below).

**Figure 1:**
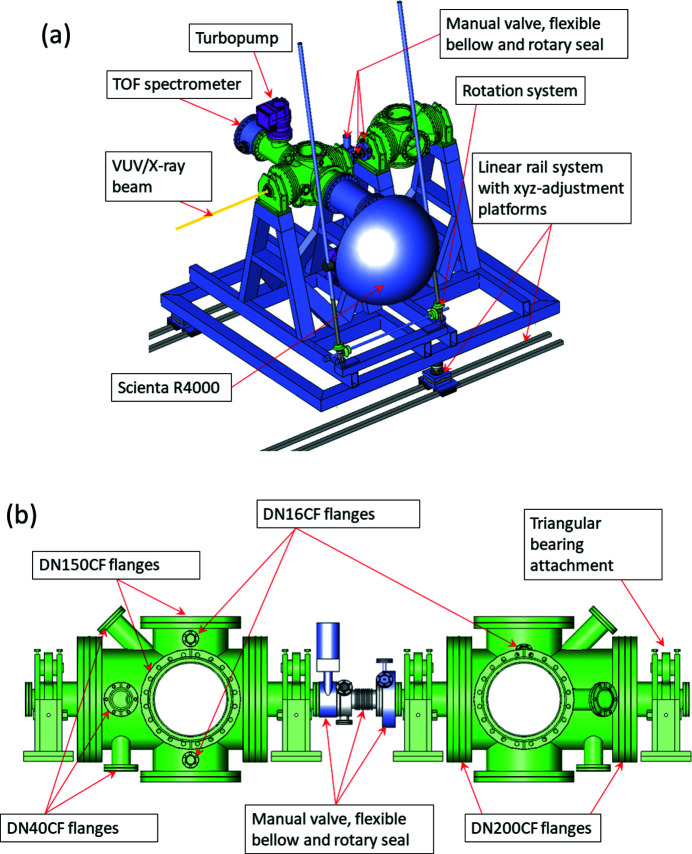
(*a*) Overview of the GPES, showing the left-hand upstream (coincidence) chamber and the right-hand downstream (user) chamber. The electron spectrometer (Scienta R4000) and ion spectrometer are mounted at their default positions in the coincidence chamber. (*b*) Sideview of both chambers and detailed description of flange sizes.The main components are labelled and indicated by red arrows.

From the beginning of the design phase, the aim was to create a versatile, modular and portable experimental configuration, which would be compatible with different sample injection systems. The starting point was a crossed-beams configuration in which a signal detection axis, flight axis of sample particles and the photon beam propagation axis cross. The endstation consists of two separate experimental chambers that include crossed-beams configurations and can be mounted after each other on a rail system, which allows movement along the beam axis [see Fig. 1 ▸(*a*)]. The movement enables either chamber to be brought into the focus of the photon beam. The upstream chamber (further referred to as the *coincidence chamber*) hosts a permanent electron spectrometer (SCIENTA R4000) and a momentum-resolving ion TOF (TOF) spectrometer [Fig. 1 ▸(*a*)] and is equipped with an inner mu-metal shielding to block out external magnetic fields. The coincidence chamber can be considered as the main installation of the GPES.

The downstream chamber (further referred to as the *user chamber*) is a replica of the coincidence chamber without mu-metal shielding and can be used to mount different users’ detectors or instruments, or additional beamline instruments. For example, a magnetic bottle type electron TOF spectrometer and a pair of cation/anion TOF spectrometers for negative-ion/positive-ion coincidence spectroscopy can be mounted in the user chamber. The magnetic bottle electron spectrometer is available to external users, presently in collaboration with the University of Oulu (Finland). The negative-ion/positive-ion coincidence instrument becomes available to external users after the completion of its commissioning.

When experiments are performed in the user chamber the beam goes through the coincidence chamber. In addition to the variable polarization of the undulator radiation, both chambers can be rotated around the photon beam axis over a 90° range. The coincidence chamber can be rotated by an integral independent support system and does not require an external crane for lifting and support [see Fig. 1 ▸(*a*)]. Both chambers have separate stands and they can be connected by means of a rotary seal and a flexible bellow that have three radial DN16CF ports for mounting different diagnostic or measurement instruments [Fig. 1 ▸(*b*)]. The chambers can also be isolated from each other by a manual gate valve.

The principal design of both chambers consists of a cylindrical main body with two DN200CF end flanges and with DN150CF four-way crosses that determine the measurement and sample injection planes [see Fig. 1 ▸(*b*)]. The distance of these DN150CF ports from the chamber axis is kept at minimum in order to retain flexibility for mounting different instruments and sample systems. In order to reduce the distance between the focal points of the upstreams and downstreams chambers and the movement needed when bringing either of the chambers to the focal position of the photon beam, the four-way DN150CF crosses are located asymmetrically depending on the centre between the two DN200CF end-ports. The focal positions of the two chambers are normally separated by 550–600 mm. The movement and alignment of the GPES is accomplished using a linear rail system, which is cast on the experimental hall’s floor and three alignment sliders [Fig. 1 ▸(*a*)].

The axes of two DN38CF ports for user equipment pass through the focal point of the chamber and intersect with the main (beam) axis at a 45° angle [see Fig. 1 ▸(*b*)]. On the plane of the DN150CF four-way cross, there is an additional DN16CF port and its axis is directed to the focal point of the chamber at 45° relative to the normals of the DN150CF ports. Additional ports (2 × DN38CF, 2 × DN16CF) can be used, for instance, for a pressure gauge, a viewport for alignment, a quartz crystal microbalance or a shutter of the sample source, respectively.

## 3. Momentum-imaging multi-particle coincidence setup   

A pulsed extraction of ions is used for the present coincidence setup. An electron detected by electron spectrometer triggers the extraction voltage pulse to the ion TOF spectrometer and the electron signal is used as an exact starting time for TOF measurements of the cations created in the initial ionization process by photons. In the following the detailed description of electron and TOF spectrometer together with the data acquisition concept for a coincidence spectroscopy is given.

### 3.1. Electron spectrometer and ion TOF mass spectrometer   

#### 3.1.1. Electron spectrometer   

The only permanently installed instrument at the GPES is a modified Scienta R4000 electron spectrometer. It is an electrostatic hemispherical electron energy analyzer with 200 mm mean radius, equipped with a five-element electrostatic lens, trajectory correction electrodes and a choice of entrance slits on movable carousel. The original CCD detector has been replaced by a fast resistive anode position-sensitive detector (Quantar Inc., Model 3395A). A three-plate (Z-stack configuration) microchannel (MCP) detector with 40 mm diameter has a spatial resolution of 0.1 mm. The readout of the detector is gathered via the Quantar Inc. type 2401B Position Analyzer ADC unit. A home-made spectrometer control software regulates the Scienta power supply modules via USB link and utilizes Scienta’s original software components for initialization and calibration tasks and for lens’ voltage tables (Mårtensson *et al.*, 1994 ▸). The readout of the electron detector is, for standalone electron spectroscopy purposes, done using the digital output signal from the Quantar’s Position Analyzer, which provides 10-bit *X* and *Y* position values for each successfully processed electron hit. The Position Analyzer also provides analog *X* and *Y* position signals that are used in data acquisition in the coincidence mode, as described in the following.

The present readout configuration is optimized for high position (and consequently energy) resolution, which is suitable for experiments with dilute targets with relatively low electron rate. The resistive anode works as a fast single-hit detector, with the dead-time mainly arising from the analog-to-digital converter (ADC) which for the 10-bit conversion is 4 µs per electron. Additional dead-time of 1–3 µs is added by the processing software reconstructing the electron image at each step of the spectral scan. In the current configuration, the saturation count-rate is about 1.5 × 10^5^ s^−1^. However, the saturation behaviour of the resistive anode detector is much more straightforward than that of the original CCD-based multi-hit detector, where complicated (and hidden-from-user) algorithms are used to evaluate true electron counts. The software for the GPES electron spectrometer incorporates a saturation correction using a single calibration parameter of detector dead-time. The saturation problems are more critical for the measurements of electron spectroscopy than for coincidence studies (Vos *et al.*, 2009 ▸). This information determines the capabilities of the modified Scienta R4000 spectrometer.

#### 3.1.2. Ion TOF spectrometer   

**Figure 2:**
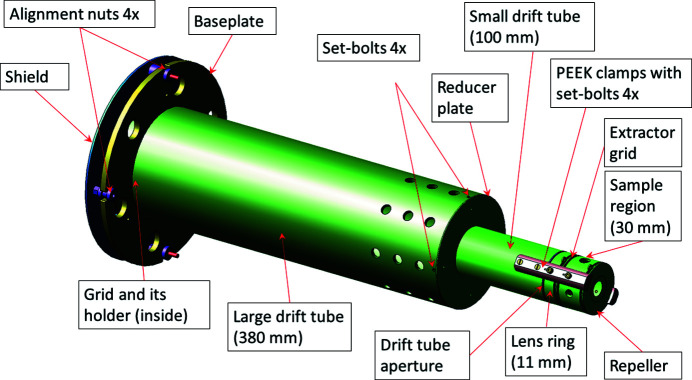
Overview of the modular TOF mass spectrometer unit. Dimensions of the corresponding modular elements on the axis of the TOF spectrometer are given in parentheses.

The ion mass spectrometer is a modified Wiley–McLaren type TOF analyzer (Wiley & McLaren, 1955 ▸), optimized for momentum imaging on the position-sensitive detector. A standard Wiley–McLaren setup would have flat-field determinations by grids, whereas the present TOF has two electrostatic lens elements, one in front of the drift tube and an additional lens element. The standard Wiley–McLaren design is not optimized for momentum imaging, whereas the present setup can be optimized for, for example, momentum imaging, geometrical source imaging or for best ion TOF resolution. The ion mass spectrometer is equipped with positively and negatively biased (by pulsed or constant voltage) repeller and extractor electrodes around the sample source region (see Fig. 2 ▸). In addition, there is an acceleration electrode at the entrance aperture of the ion drift tube. In order to maximize ion detection efficiency, only the extractor electrode is equipped with a grid preventing the ion acceleration field from penetrating into the source region, which would be detrimental for the electron energy resolution in coincidence measurements. The aperture of the acceleration electrode acts as an ion lens. To modify this lens effect without changing the total accelerating voltage, an additional lens electrode (‘lens ring’ in Fig. 2 ▸) was added. It can be used to optimize the momentum imaging performance and to adjust the detector image size for ions with a given initial velocity.

The TOF spectrometer comprises modules, so that a source region with a different design can be attached to the standard drift tube module, or both can be replaced while retaining the standard detector module.

A Roentdek detector of 80 mm active diameter MCP together with Hex-anode delay-line setup was chosen to satisfy the demands for advanced ion detection tasks like large area imaging, precise multi-hit timing information and high detection rate. The readout scheme of the detector – a three-layer hexagonal delay-line anode – enables the detection of multi-hit events as long as two particles do not arrive at the same time and at the same position, with limits determined by the electronic dead-time and anode size (Jagutzki *et al.*, 2002*a* ▸,*b* ▸). The position in the hexagonal coordinate frame (three coordinates) is determined by the differences of the arriving times from the signals and can be transformed into a Cartesian coordinate system (two coordinates). The redundancy of position and timing equations improves the detection efficiency and the analysis of multi-hit events. The capability of the simultaneous detection of the multi-hit events is especially valuable for ion–ion coincidence experiments, during which multiple fragments with the same mass-to-charge ratios (or similar TOFs) are often created.

In order to maximize the data acquisition speed a high-bandwidth waveform signal is transferred from the ion detector to two fast sampling ADC units (proprietary fADC4 cards by RoentDek Handels GmbH) without external signal processing electronics except a preamplifier. Fast recording (1.25 GHz) of raw signal waveforms allows the greatest possible flexibility in post-processing and software-based signal analysis and filtering. The *CoboldPC* program package (from RoentDek Handels GmhH) is used to read out the digital data from the delay line detector and online analysis of the raw data.

Geometrically, the TOF spectrometer is composed of two successive cylinders with outer diameters 110 mm and 50 mm in front of the Roentdek detector system (see also Fig. 2 ▸). The smaller cylinder (50 mm) contains repeller and extraction plates separated by 20 mm and followed by the focusing lens ring. The cylindrical drift tube has two parts: a short tube with 50 mm diameter and 100 mm length is connected to a larger tube with 110 mm diameter and 500 mm length. The larger drift tube cylinder is attached to the base plate in front of the MCP detector.

The TOF spectrometer has four elements with adjustable potentials – the ion repeller and the extractor grid, the aperture (‘lens’) in front of the drift tube and the entire drift tube assembly together with its entrance aperture (Fig. 2 ▸). In standard operation, the ion source position along the spectrometer’s axis (where the radiation passes the sample region) is kept at the ground potential and positive and negative voltage pulses of the same magnitude are applied to the repeller and extractor (the ‘extraction voltage’). The final energy of the ions in the drift tube is chosen by the drift tube potential (the ‘acceleration voltage’) and the TOF resolution can be optimized by choosing a suitable ratio of the extraction and acceleration voltages, with only a minor effect from the lens voltage. The optimization can be made to satisfy the Wiley–McLaren condition (Wiley & McLaren, 1955 ▸), whereby the influence of the actual ion source size (spread along the TOF axis) on the TOF resolution is minimized. The lens voltage primarily affects the trajectories of the ions, the lateral magnification of the source and the imaging of the initial momenta of the ions on the detector. The lateral spread of the trajectories of ions with large initial kinetic energy can exceed the diameter of the MCP detector, in which case the ion collection angle is less than 4π sterad and the momentum image becomes clipped. In the present geometry (Fig. 2 ▸) without the effect of the lens element, the maximum kinetic energy of ions that are fully detectable by the 80 mm-diameter MCP detector can be estimated as
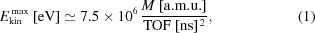
where *M* is the mass of ion in atomic mass units (a.m.u.)] and TOF is its flight time. The lens voltage, however, can be used to adjust the ion trajectories and the size of the momentum image, while keeping the desired TOF range of the ion spectrum.

Initial values for the suitable TOF spectrometer’s voltage settings can be obtained by numerical simulations of the spectrometer, which also provide useful insight into the operational conditions in the pulsed-field coincidence mode. For the measurements reported here, we modelled the ion flight trajectories of N^+^ ions with the initial kinetic energy equal to 4 eV – a typical value obtained in the dissociation of 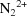 (Pandey *et al.*, 2016 ▸). The electric fields in a cylindrically symmetric grid were calculated using home-made software developed in the *Igor Pro* software environment (WaveMetrics Company) and the ion flight trajectories were then traced through the grid.

First, a acceleration voltage satisfying the Wiley–McLaren space focusing condition and the optimal TOF resolution (Wiley & McLaren, 1955 ▸) was found for the the 200 V extraction voltage (±100 V across the sample region) by using ions trajectories starting from slightly different positions (±5 mm) from the nominal source point in the middle of the sample region. The ions had no initial kinetic energy. The lens voltage was chosen to keep uniform electric field in the acceleration region in front of the drift tube, thus minimizing the effect of the lens. The optimal acceleration voltage for the best TOF resolution was found to be −1240 V.

**Figure 3:**
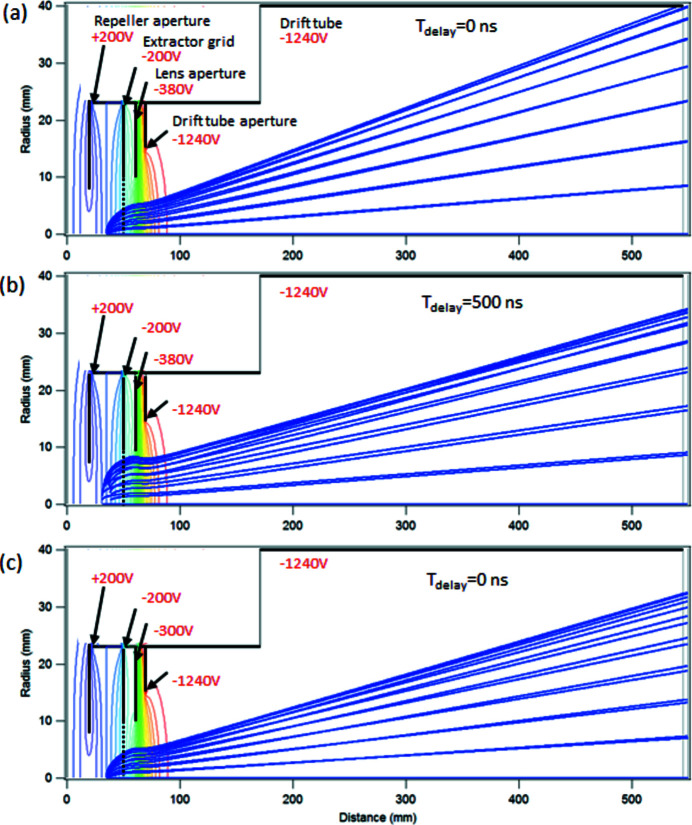
Numerical simulations of ion trajectories (blue lines) in the ion TOF spectrometer, varying the angle of their initial momentum from the *x*-axis. The cross-cuts of the cylindrically symmetric spectrometer’s components are shown by black lines, labelled (see also Fig. 2 ▸), and the applied voltages shown. The multicoloured curves represent isopotential surfaces, from red (most negative) to violet (most positive) values. Panel (*a*): trajectories of the N^+^ atomic ions with 4 eV initial kinetic energy, created in a point source. Voltages are optimized for best momentum imaging (see text for details). Panel (*b*): same conditions as for panel (*a*) with the added extraction pulse delay of 500 ns. Panel (*c*): same conditions as for panel (*a*), except using the lens voltage corresponding to the settings in the reported experiment in Figs. 5 ▸ and 6 ▸.

Then, the momentum imaging conditions were optimized by varying the lens voltage. Fig. 3 ▸ shows the results of three simulations, with the trajectories of individual lines shown as blue lines. The ions for these simulations were generated from a point source in the middle of the sample region with the initial kinetic energy of 4 eV and with the different trajectories corresponding to different angles θ between the initial momentum vector and the TOF axis. Fig. 3 ▸(*a*) corresponds to the conditions where ions emitted at ±θ are detected at the same radius, giving the best resolution for determining the transverse momentum component from the image. The lens voltage for this condition was −380 V.

The above simulation does not take into account the delay between the creation of the ions and the application of the extraction pulse. In the coincidence mode where the electron provides the trigger for applying the extraction pulse, the contributions to the delay are: (i) the electron flight time in the lens system of the electron spectrometer, (ii) electron flight time in the hemispherical analyzer and (iii) delays in the electronic circuit. The first depends on the electron kinetic energy, the pass energy and various lens voltages, and the second is determined only by the pass energy. Low kinetic and pass energy settings cause longer pulse delays. The electronic contribution presently forms (for common pass energy and electron kinetic energy ranges of 10–100 eV) approximately two-thirds of the total delay that is of the order of 500 ns. This *T*_d_ = 500 ns delay was applied in the second simulations shown in Fig. 3 ▸(*b*), where closer inspection shows that the ions now originate from a circle around the nominal source point (elongated along the *y*-axis since the radial coordinate is shown in a larger scale). The radius of this circle is *R*_d_ = (2*E*_kin_/*M*)^1/2^ *T*_d_, the spread from the point source in an initially field-free sample region by the time the extraction pulse is applied. If *R*_d_ becomes comparable with the dimensions of the sample region, the effect on the ion trajectories is significant, as seen in Fig. 3 ▸(*b*). The momentum image becomes considerably compressed and also the momentum resolution deteriorates.

The simulation shows that the effect of the extraction pulse delay in a pulsed-field coincidence setup cannot be ignored. The delay affects the detector image more, whereas the TOF spectrum is not sensitive to it and is more pronounced at low electron kinetic and/or pass energy settings. The influence of the pulse delay diminishes rapidly with the decreasing initial velocity of the ions, and is generally negligible for heavier (*M* ≳ 30) ions. In order to minimize the total delay time in the present setup, future upgrades with faster electronic components are planned.

Finally, Fig. 3 ▸(*c*) shows the simulation where the lens voltage value was optimized empirically in the experiment, by obtaining the momentum image with the best contrast. The value of −300 V was obtained and used also for the measurements reported below (Figs. 5 and 6). The simulation demonstrates the effect of adjusting the size of the momentum image by the relatively small variations of the lens voltage. Why the experimental optimization did not arrive at exactly the simulated optimum lens settings can be due to several reasons: the chosen voltage could partly compensate for the distortions due to the pulse delay, the smaller momentum image could exhibit sharper features even if the actual momentum resolution is not improved and, finally, the accuracy of the experimental optimization was limited.

### 3.2. Data acquisition concept for coincidence experiments   

**Figure 4:**
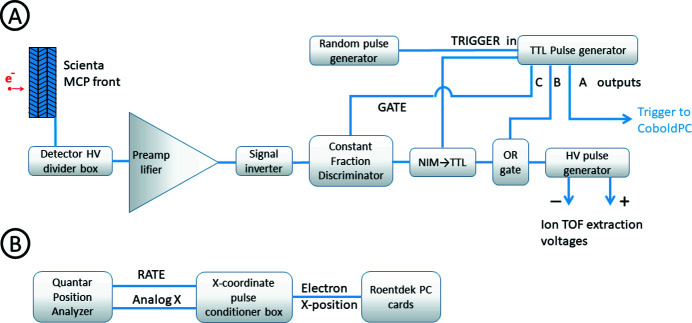
Signal triggering and processing schemes. Panel (A): signal conditioning for the coincidence and artificial/random signal triggering. Panel (B): signal processing for electron energy recording.

In this section the main elements of a data acquisition and triggering scheme are described (see also Fig. 4 ▸). The lens system, analyzer and MCP detector with a resistive anode of the Scienta R4000 electron spectrometer are biased by the voltage rack of the modified Scienta spectrometer. A four-channel programmable constant high voltage supply (CAEN NDT1470) provides voltage settings for the MCP front, drift tube, lens element and delay-line Hexanode of the TOF spectrometer. The extraction field pulses for ions are generated by a DEI PVM-4210 HV (high voltage) pulse generator, providing an equal pulse of negative and positive polarity for the extractor and repeller electrodes, respectively. The HV pulses are triggered by a TTL pulse generator (Quantum Composer 9530), while the TTL pulse generator can be triggered in a variety of modes. Different predefined optimized triggering settings (for the regular TOF measurements, for the coincidence measurements, for the scaling voltages of TOF spectrometer) can be saved to and retrieved from the pulse generator’s control board. In the case of the regular ion TOF measurements triggering is determined by the certain constant pulse frequency of the TTL pulse generator.

Regular ion TOF measurements are performed at fixed frequency (up to ∼20 kHz) of extraction pulses. Two TTL output channels are in use [see Fig. 4 ▸(*a*)]. The first one, output A, is a short pulse that is converted to a nuclear instrumentation module (NIM) signal by a logic converter (ORTEC 9307) and it triggers the waveform acquisition cards in the Roentdek computer. The second one, output B, from the TTL generator triggers the HV pulse and determines the duration for which the extraction field is present in the ion source region.

In the case of coincidence measurements the ion extraction HV pulse must be synchronized with the arrival time of the electrons which is provided by the electron detector of the Scienta R4000 electron spectrometer. In order to retrieve the kinetic energy distribution of detected electrons from each coincidence event the electron hit position on the axis of the detector in the energy-dispersive direction must be saved for each trigger separately. The setup utilizes a Roentdek waveform capture input channel that is not required to decode the ion hit position from the delay-line Hexanode. The electron-dispersive position (kinetic energy) information from the analog output of the Quantar position analyzer is forwarded to that input channel of the Roentdek and the position value is recorded as the (maximum running average of the) trace height for each triggered acquisition [see Fig. 4 ▸(*b*)]. If the coordinate voltage value is close to zero, it means no valid electron position was available from the position analyzer. This situation corresponds to either a generated constant-frequency trigger (as opposed to electron trigger) or a fault in encoding the electron position from the resistive anode.

In a pulsed-mode coincidence setup, a common problem is the separation of true and false coincidences, the latter arising when the ions recorded in a coincidence event in fact originated from different quantum events than the electron triggering the acquisition – such as from different molecules or particles. Although the true and false coincidences cannot be distinguished in an event-by-event basis, statistically a subtraction of a false coincidence background can be made from various distributions derived from the coincident dataset (Prümper & Ueda, 2007 ▸). For this purpose, the setup allows alternating or even mixing of electron and constant-frequency triggers, the latter representing the false coincidences. Although there is no specific input for the *CoboldPC* software to distinguish between the true (electron) and false (constant-frequency) triggers, the level of the electron position input conveniently also contains that information (false trigger = near-zero signal).

The data transfer and processing sequences of electron signals are illustrated in Fig. 4 ▸. The upper panel (A) describes the signal conditioning for the electron triggers and the application of the HV extraction pulses, and the bottom panel (B) corresponds to the signal transfer and recording of electron energy values. In panel (A), the shaping of the various triggers is done by the TTL pulse generator. The output channel A provides a short pulse as start trigger for the ion TOF measurement and channel B provides a long (several µs) pulse enabling the HV extraction voltage during the TOF acquisition time. Each HV extraction pulse is accompanied by strong electromagnetic noise picked up by the electron detector, which can result in false triggers. The output channel C provides a pulse that is by 1–2 µs longer than that from channel B; it is used to gate the constant fraction discriminator (CFD) unit preventing it from relaying these false-noise-generated pulses as new triggers. The purpose of the OR-gate is to remove the TTL pulse-generator-induced delay from the output of the HV pulse, by first bypassing the TTL generator with a short trigger pulse directly to the HV pulse generator and, while this pulse is still high, combining it with the channel B output. Finally, a separate home-made pulse generator (‘random pulse generator’) provides alternative triggers to the TTL pulse generator, mixed with the electron triggers. These are used for the removal of false coincidences as described above.

## 4. Commissioning results   

### 4.1. Case I: molecular nitrogen   

**Figure 5:**
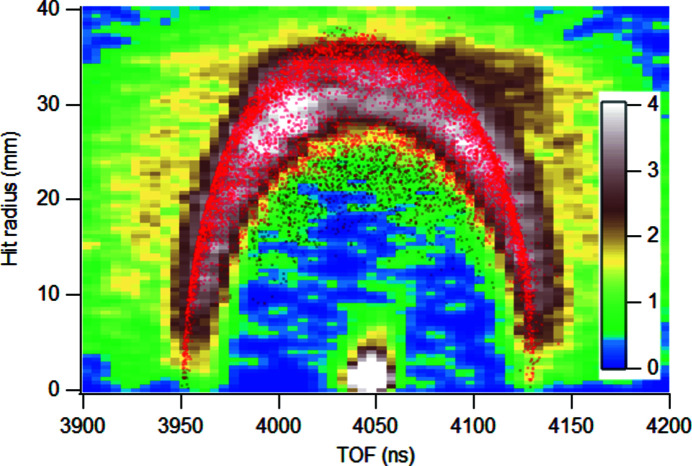
False-colour intensity map of N^+^ ions detected in coincidence with N 1*s* photoelectrons, depicting the ion hit radius on the detector as a function of the ion’s TOF. Red dots are a result from Monte Carlo ion trajectory simulation for 3.5 eV kinetic energy N^+^ ions.

The Coulomb explosion process of molecular nitrogen, N^2+^ → N^+^ + N^+^, following N 1*s* ionization and Auger decay, was chosen as a test case for the ion spectrometer’s momentum imaging capability in coincidence mode. An electron–ion–ion coincidence (PEPIPICO) data set was recorded using 442 eV photons and operating the electron analyzer at 100 eV pass energy with the detected kinetic energy window centred at 31 eV. The spectrometer’s entrance slit was set to the largest possible value (resolution is not the limiting factor in this experiment). The TOF and hit positions (*X*, *Y*) of nitrogen ions on the detector were recorded following the detection of N 1*s* photoelectrons that triggered the ion extraction high-voltage pulse. The ion spectrometer was operated at the following voltages: *U*_source_ = ±200 V, *U*_lens_ = −300 V, *U*_drift_ = −1240 V. Fig. 5 ▸ shows a false-colour plot of all the recorded N^+^ ions as their hit radius *R* on the detector versus TOF. The saturated bright spot at around 4050 ns arises from slow, doubly ionized parent molecules. The spot was used to correct the true centre of the ion image on the detector before obtaining *R*.

Fig. 5 ▸ shows the experimental raw data for calculating the ion momentum vectors and kinetic energies. For the calculation, conversion coefficients *c*_⊥_, *c*_∥_ for *v*_⊥_ = *c*_⊥_ *R* and 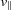 = 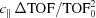 (where TOF_0_ is the flight time of the ions with no initial velocity) must be known. These coefficients were obtained from ion flight trajectory simulations such as presented in Fig. 3 ▸.

The red dots in Fig. 5 ▸ represent the result of such a Monte Carlo simulation of 10000 ion trajectories, where the N^+^ ions had the initial kinetic energy of 3.5 eV with the initial velocities distributed isotropically. In addition, a finite source volume was introduced as a Gaussian distribution with σ = 2 mm and cutoff at 5 mm from the centre, both in the longitudinal and radial directions. In the latter, the ion distribution in the source was weighted, by ∝ *R*, to account for the cylindrical symmetry’s volume effect. For the final simulation, an extraction pulse delay of 500 ns was introduced for better correspondence with the experimental conditions. In Fig. 5 ▸, its effects can be seen as a slight flattening of the top part of the simulated distribution. A corresponding flattening can be seen in the experimental distribution. The reason is that from the source, expanded during the delay [see Fig. 3 ▸(*b*)], the ions with high radial velocity pass close to the edge of the aperture of the lens element, where electro-optical aberrations are strong.

**Figure 6:**
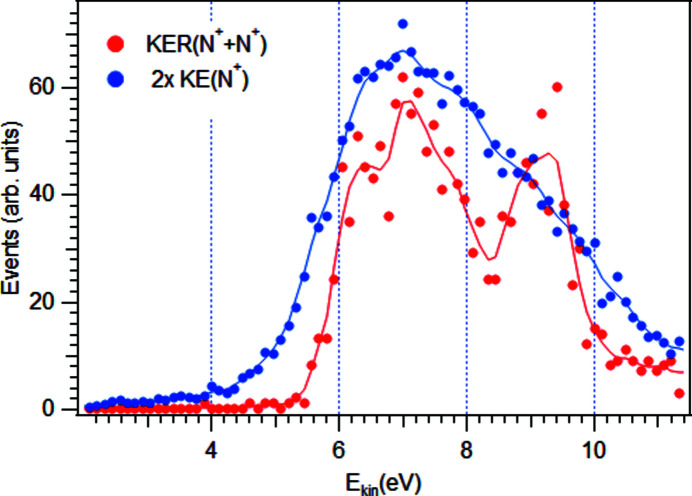
Measured kinetic energy release (KER) in the dissociation of 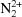, following 1*s* core ionization and Auger decay. The blue markers show the KER distribution obtained from doubled single-ion kinetic energies [2 × KE(N^+^)] and the red markers show the KER as a sum of the energies of two ions detected in coincidence.

After determining the momenta of the ions, kinetic energy release (KER) in the charge-symmetric dissociation of molecular nitrogen dications, 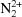 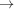 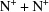, was determined. Fig. 6 ▸ shows the KER distributions obtained by two different methods: (i) estimated from the kinetic energy (KE) distribution of all individual N^+^ ions detected, by doubling the energy values, and (ii) through coincidence analysis of N^+^ ion pairs, as a distribution of the sum of their kinetic energies. As the figure shows, the latter method yields a considerably better resolved KER distribution, since only ions that form true coincidence pairs can be chosen by checking that their momentum vectors are antiparallel as required by momentum conservation. The first method can give a rough estimate for KER in a faster experiment with trigger rates too high for a coincidence analysis.

The presented KER curves are in a good agreement with the earlier study of electron-impact dissociative double ionization of N_2_ (Pandey *et al.*, 2016 ▸).

### 4.2. Case II: thiophene – medium-sized molecule   

A demonstration of a coincidence measurement of a core ionized medium-sized organic molecule is shown in the example of thiophene – a molecule exhibiting a rich dissociation landscape with well defined features in the corresponding ion–ion coincidence map (Kukk *et al.*, 2015 ▸). Thiophene is an excellent model system due to its rigid aromatic ring structure, lack of side chains and of room-temperature isomers. An Auger Electron Photo–Ion Photo–Ion Coincidence (AEPIPICO) experiment was performed by ionizing the thiophene molecules with monochromated radiation of 190 eV photon energy, tuned to efficient sulfur 2*p* core ionization. The electron spectrometer was operated at 200 eV pass energy with 1 mm entrance slit providing electron energy resolution of about 0.77 eV (FWHM). The centre of the kinetic energy window was set at 138 eV to capture the S LVV Auger electrons, and the width of the energy window at the given pass energy was 12 eV. The voltage settings of the ion TOF spectrometer *U*_source_ = ±200 V, *U*_lens_ = −400 V, *U*_drift_ = −1240 V were used.

The sulfur 2*p* vacancy is, in a few femtoseconds after the core ionization, filled by the Auger process and an Auger electron is ejected. The resulting doubly charged molecule dissociates into two charged fragments that can be detected in coincidence with the emitted Auger electron.

**Figure 7:**
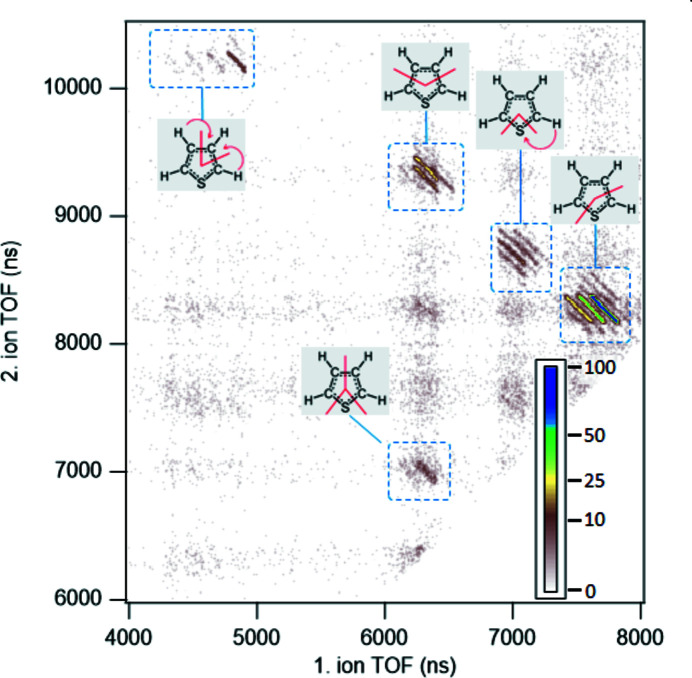
Ion–ion coincidence map of the thiophene molecule, measured in coincidence with the sulfur LVV Auger electrons (AEPIPICO) following S 2*p* core ionization with 190 eV photons. Identification of the main groups as various dissociation channels, according to Kukk *et al.* (2015 ▸), is shown. The colour scale is set to emphasize the weaker features on the map.

Fig. 7 ▸ shows an ion–ion coincidence map as a summary of the measurement, demonstrating the ability of the coincidence setup to resolve the details of the coincidence patterns using the TOF information only. The individual tilted lines on the map correspond to dissociation channels with specific end products; for example the strongest line centred at about 7700 ns (1st ion), 8300 ns (2nd ion) on the map arises from the two-body process C_4_SH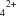 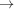 C_3_H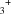 + CSH^+^. The negative slope of −1 arises because of the momentum conservation in the dissociation. The other, weaker, lines in a group are due to hydrogen loss and/or migration, but all correspond to the same splitting pattern of the thiophene ring as illustrated in the figure. Different ring splitting patterns give rise to other groups seen on the map. A detailed analysis of the photofragmentation pathways, hydrogen dynamics and internal energy dependence on the thiophene dissociation has been published by Kukk *et al.* (2015 ▸).

The data shown in Fig. 7 ▸ are raw coincidence data in the sense that no false coincidence background has been subtracted. The false coincidences where ions in a pair originate from different molecules do not exhibit any momentum correlation and give rise to the weak diffuse background in Fig. 7 ▸. In the present measurement, the electron detection rate was 25–30 s^−1^, which under the given settings of the electron spectrometer resulted in a satisfactory ratio of true-to-false coincidences. The ratio can be improved further by reducing the electron detection rate by, for example, lowering the photon flux. False coincidence subtraction can be performed, if necessary, by using artificially generated triggers as outlined in Section 3.2.

Each ion pair in the full dataset is associated with a detected Auger electron at a certain kinetic energy. Thus, coincident ion and ion-pair yields can be extracted as a function of the Auger electron energy, which provide valuable information on the dependency of the dissociation patterns on the internal energy and the electronic configuration of the parent molecular dication (Kukk *et al.*, 2015 ▸). In addition, each detected ion has the information on its momentum vector, constructed from the hit position on the detector as well as from the ion’s TOF. This enables to (i) perform additional filtering of true coincidences by applying the requirement of momentum correlation, and (ii) follow the changes of the ion momenta and the KER in the dissociation reactions as a function of the Auger electron energy.

In view of the results of coincidence studies of medium-sized molecules such as thiophene, the extension of coincidence spectroscopy to more complex and even heterogeneous molecular systems, such as hydrated clusters of biomolecular building blocks, appears promising.

### 4.3. Case III: angle-resolved Ar 3*p* photoionization   

The vacuum chamber of the GPES can be rotated in the dipole plane along the photon beam [see Figs. 1(*a*) and 1 ▸(*b*) ▸]. Angular distributions of photoelectron and Auger electron lines can thus be determined by measuring the electron spectra at different angles, while fixing the direction of the electric vector of linearly polarized light. There are, however, clear advantages in performing such measurements in the opposite way: the electron analyser is not moved, but instead the direction of linear polarization is changed. In that way, the electron analyser will detect at the same source volume, which can greatly diminish intensity variations in the electron spectra and increase reliability of the results. This works, of course, only if the light spot in the experiment does not shift upon the change of the polarization, which is in practice done by changing the phase of the magnetic arrays in the undulator.

**Figure 8:**
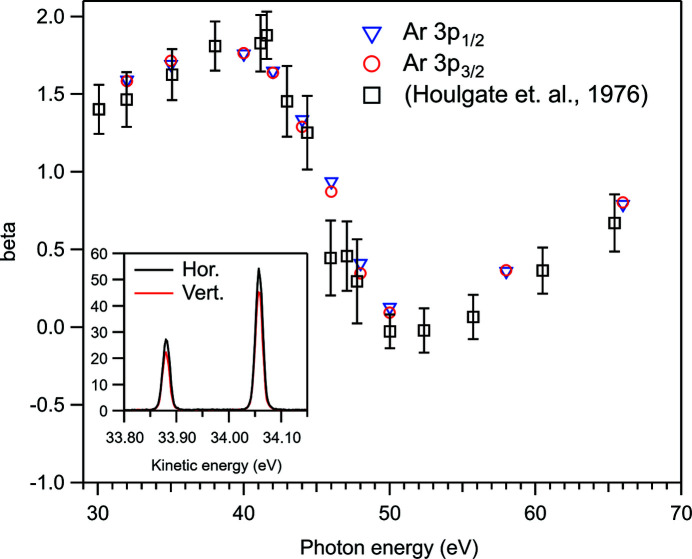
The asymmetry parameter, β, of the Ar 3*p* photoelectron lines as a function of photon energy. The open squares with error bars show the earlier results (Houlgate *et al.*, 1976 ▸). The inset shows the normalized Ar 3*p* photoelectron lines at the photon energy of 50.0 eV with horizontal and vertical linear polarization.

The suitability of the GPES at the FinEstBeAMS for angle-resolved photoelectron spectroscopy was tested by measuring the Ar 3*p* photoelectron lines both with horizontal and vertical linear polarization while keeping the electron analyser in the horizontal direction (0°). The spectra at each photon energy were recorded immediately after each other by only changing the direction of the polarization and the undulator gap in between the measurements, *i.e.* the settings of the monochromator were not touched. No kinetic energy shift was observed in any of the measurements within the accuracy of Gaussian fitting; a shift could have indicated a movement of the photon beam upon the change of the polarization. A pass energy of 10 eV was used in the measurements. The inset of Fig. 8 ▸ shows an example of the measured spectra after normalization to the photodiode current and pressure. Starting from the general formula for the differential photoionization cross section (Cooper & Zare, 1968 ▸),
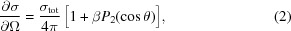
the asymmetry parameter, β, can be obtained from such measurements using the equation
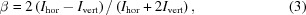
where *I*_hor_ and *I*_vert_ are the intensities (areas) of the photoelectron lines measured with the horizontal and vertical linear polarization, respectively. Our results for β are compared with those of Houlgate *et al.* (1976 ▸) in Fig. 8 ▸. The datasets agree with each other within the error bars of Houlgate *et al.* (1976 ▸) apart from the photon energy of 46 eV, where the β value of Houlgate *et al.* (1976 ▸) appears anomalously low. The uncertainty of our values due to the fitting of the spectra is <±0.02 units for the 3*p*_1/2_ photoelectron line and <±0.012 units for the 3*p*_3/2_ photoelectron line. These uncertainties are within the sizes of the symbols in Fig. 8 ▸. There can be an additional error due to the incomplete linear polarization of the incident light, but the present data do not allow us to quantify the degree of linear polarization because the β parameter of the studied photoionization channels is not known exactly at any of these photon energies. Nevertheless, the good agreement with the previous results indicates that angle-resolved electron spectroscopy can be conducted in the described way.

### 4.4. Case IV: total electron yield measurements of the solid transition metal samples   

**Figure 9:**
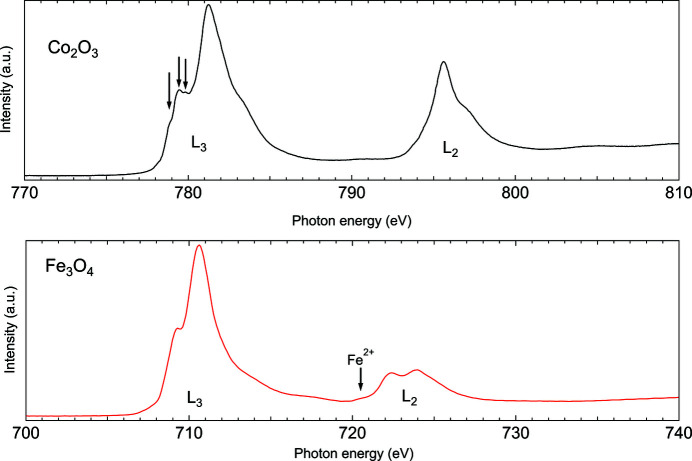
Example of total electron yield measurements at the GPES. The near-edge absorption fine structure of the solid Co_2_O_3_ and Fe_3_O_4_ samples in the photon energy range 700–810 eV is presented. For Co_2_O_3_, arrows indicate the several minor spectrally resolved components in the Co^3+^ 2*p*3*d* octahedral ligand field multiplet.

In addition to spectroscopic measurements in the gas or liquid phase, reference signals or spectra from solid matter can be recorded at the GPES. For example, commissioning results of total electron yield measurements of the solid transition metal samples Co_2_O_3_ and Fe_3_O_4_ at the Co and Fe *L*_2,3_-absorption edges are presented in Fig. 9 ▸. The X-ray absorption spectra displayed in Fig. 9 ▸ give examples of reasonable statistics at resolution sufficient to resolve, for example, the relatively rich multiplet structure to an extent that unambiguous determination of homogeneous (Co_2_O_3_) or mixed (Co_3_O_4_) charge state is possible (Šutka *et al.*, 2016 ▸), respectively. A great majority of physico-chemical and biological processes take place in a liquid environment. The interactions of metals with biologically important molecules like amino acids (Remko & Rode, 2006 ▸), nucleobases (Lynam, 2008 ▸) or, more specifically, neurotransmitters (Snoek *et al.*, 2003 ▸; ÇarÇabal *et al.*, 2005 ▸) form a significant part of these processes. Transition metals are essential for numerous biochemical processes. Transition metal compounds and their free and adsorbed nanoparticles are potentially interesting samples for studies at the GPES. The present case demonstrates a possibility to compare the electron spectroscopic results of the diluted samples with data recorded from solid state samples in the same beam time.

## 5. Sample introduction systems and setups   

Each specific form of samples, such as atoms, molecules, clusters, nanoparticles, aerosols in the gas phase or in liquid environment, may need its own sample delivery configuration into the experimental vacuum chamber. In the following, the most basic configurations are described.

### 5.1. Needle inlet system   

This system is the simplest way to introduce atomic or molecular samples with high vapour pressure to the interaction region of the coincidence setup. A sample tube is attached via a port aligner (edge welded bellow with three adjustment studs) to a DN150CF inlet flange. The sample is introduced via needle at the end of 6 mm stainless steel tube. The length of the presently used needle is around 50 mm, inner diameter 110 µm and outer diameter 0.25 mm. The cross section of the needle is chosen as small as possible in order to avoid distortions of electric fields in the interaction region of spectrometers. The minimized gas conductance of the inlet needle also moderates pressure fluctuations in the experimental chamber. However, it is possible to replace the needle by a wider one if it is necessary due to low vapour pressure of the sample. The sample pressure in the vacuum chamber can be adjusted by a gas inlet valve between the inlet system and sample gas container.

### 5.2. Modular (differential pumped) sample introduction systems   

The injection systems for more complex quantum systems, clusters, nanoparticles, aerosols or liquids, need multicomponent vacuum setups. One of the most crucial demands is the protection of the MCP detectors of the spectrometers from the destructive influence of high sample gas pressure, especially in high voltage conditions. In other words, cluster sources, aerodynamic lens systems for nanoparticles (or aerosols) and liquid microjet setups need an expansion chamber and (turbo)pumps with a pumping speed for high gas load (at least 2000 l s^−1^). The expansion chamber needs to be connected to the experimental section through gas flow limiters such as apertures, flow tubes or skimmers. The coincidence chamber of the GPES has two opposite DN150CF flanges on the axis that is perpendicular to the axis of the electron and ion TOF spectrometers’ mounting flanges. One of these flanges can be used for mounting an expansion chamber with a gas flow limiter and specific user determined sample source. At the moment the GPES is equipped with a DN150CF five-way cross with a liquid-nitrogen trap and three HiPace 700 (Pfeiffer) pumps to enable a pumping speed of more than 2000 l s^−1^ of the sample sources with high gas load. This pumping speed is satisfactory for most well constructed liquid microjet (Riley *et al.*, 2019 ▸; Seidel *et al.*, 2017 ▸; Brown *et al.*, 2013 ▸) and cluster injection systems (Lindblad *et al.*, 2013 ▸; Kim *et al.*, 1996 ▸, 2000 ▸) keeping pressure in expansion section in the range of 10^−4^ mbar, and allowing pressures of 10^−6^–10^−7^ mbar in the experimental section.

## 6. Conclusions   

We have presented the gas-phase endstation with multi-coincidence setup at the FinEstBeAMS beamline in the MAX IV 1.5 GeV storage ring. The experimental configuration consists of the Scienta R4000 electron spectrometer and ion TOF spectrometer with a Roentdek delay line detector. A detailed description of the functional design of the endstation together with overview of the data acquisition concept has been given in this paper.

The GPES enables users to study different samples in the gas phase such as atoms, molecules, microclusters and liquids. Electron, ion and coincidence spectroscopy studies can be executed in a broad photon energy range from 4.4 eV up to 1000 eV. The configuration of three perpendicular axes in the experimental chambers enables users to mount different sample inlet systems easily. In this way the electron spectrometer of the GPES can be used even for experiments of nanoparticles and liquid samples. The first test measurements of N_2_ and thiophene (C_4_H_4_S) molecules with the multi-coincidence setup at the GPES demonstrated the possibility to use the GPES in many-particle coincidence spectroscopy. The measurements of Ar 3*p* photoelectron spectra with linear horizontal and vertical polarization show that the setup can be used to perform angle-resolved experiments. We have also demonstrated the possibility to compare the electron spectroscopic results of diluted samples with the solid targets in the case of Co_2_O_3_ and Fe_2_O_3_ at the Co, Fe *L*_2,3_-absorption edges in the same experimental beam time.
